# Local Sources of Protein in Low- and Middle-Income Countries: How to Improve the Protein Quality?

**DOI:** 10.1016/j.cdnut.2023.102049

**Published:** 2023-12-21

**Authors:** Nitya Vissamsetti, Mackenzie Simon-Collins, Sheryl Lin, Sulagna Bandyopadhyay, Rebecca Kuriyan, Wilbert Sybesma, Daniel Tomé

**Affiliations:** 1 Department of Biochemistry and Molecular Biology and Center for Physics of Evolving Systems, University of Chicago, Chicago, IL, United States; 2 Division of Reproductive Sciences and Women’s Health Research, Department of Gynecology and Obstetrics, Johns Hopkins University School of Medicine, Baltimore, MD, United States; 3 Department of Biology, Johns Hopkins University, Baltimore, MD, United States; 4 Division of Nutrition, St. John's Research Institute, St. John's National Academy of Health Sciences, Bangalore, India; 5 Yoba for Life Foundation, WB Amsterdam, The Netherlands; 6 Université Paris-Saclay, AgroParisTech, INRAE, UMR PNCA, Paris, France

**Keywords:** protein, alternative protein, digestibility, antinutrients, malnutrition, LMIC, local sourcing, protein processing

## Abstract

Protein inadequacy is a major contributor to nutritional deficiencies and adverse health outcomes of populations in low- and middle-income countries (LMICs). People in LMICs often consume a diet predominantly based on staple crops, such as cereals or starches, and derive most of their daily protein intakes from these sources. However, plant-based sources of protein often contain low levels of indispensable amino acids (IAAs). Inadequate intake of IAA in comparison with daily requirements is a limiting factor that results in protein deficiency, consequently in the long-term stunting and wasting. In addition, plant-based sources contain factors such as antinutrients that can diminish protein digestion and absorption. This review describes factors that affect protein quality, reviews dietary patterns of populations in LMICs and discusses traditional and novel small- and large-scale techniques that can improve the quality of plant protein sources for enhanced protein bioavailability and digestibility as an approach to tackle malnutrition in LMICs. The more accessible small-scale food-processing techniques that can be implemented at home in LMICs include soaking, cooking, and germination, whereas many large-scale techniques must be implemented on an industrial level such as autoclaving and extrusion. Limitations and considerations to implement those techniques locally in LMICs are discussed. For instance, at-home processing techniques can cause loss of nutrients and contamination, whereas limitations with larger scale techniques include high energy requirements, costs, and safety considerations. This review suggests that combining these small- and large-scale approaches could improve the quality of local sources of proteins, and thereby address adverse health outcomes, particularly in vulnerable population groups such as children, adolescents, elderly, and pregnant and lactating women.

## Introduction

Malnutrition and food insecurity in low- and middle-income countries (LMIC) has reincreased, despite previous decades of successful interventions and efforts that reduced its prevalence [1,2]. Around one-third of the global population is malnourished, including people with wasting, stunting, and overweight [3]. The FAO of the United Nations projects ≥600 million people in hunger by 2030 [3]. The WHO estimates >2.4 billion people do not have regular access to nutritious food [3]. The COVID-19 pandemic in 2020 and the Ukraine war have increased hunger in comparison with prepandemic levels, increases that have persisted globally through 2022 [3].

Malnutrition includes undernutrition, micronutrient deficiencies, stunting, wasting, as well as overnutrition, overweight and obesity [1]. Malnutrition can be caused by a lack of overall calories or a lack of specific nutrients in the food including micronutrients (vitamins and minerals) as well as macronutrients, particularly proteins. LMIC populations typically base their diets mainly on food staples, such as cereals and starches accounting for 49% of the diet [4]. These staple crops may not provide all necessary nutrients, increasing the risk of protein inadequacy [1]. Mid-20th century nutritional research focused on protein deficiencies until losing support to research on vitamins, which dominated the nutritional sphere until the “pendulum” swung back to protein deficiencies again [5]. The United Nations published reports such as the *International Action to Avert the Impending Protein Crisis,* which warned of a “protein gap,” a failure to meet protein requirements [5]. Several randomized clinical trials in LMIC have demonstrated the link between protein deficiency and stunting by improving child growth and birth weight outcomes with protein supplementation [[6], [7], [8]]. Many of these trials were based on animal source foods (ASFs), such as eggs and milk; thus, any shift of animal source protein toward plant-based protein should be carefully considered to safeguard consistent protein quality and nutritional outcomes.

Since then, progress has been made, but protein insufficiency and related health challenges remain a major public health issue [1,5]. This review describes factors associated with protein quality, dietary patterns of populations in LMIC, factors that can reduce the quality of protein in plant-based foods, and techniques that can be used to improve protein quality. The review considers information in the context of techniques and policies that can be implemented in LMIC to reduce protein deficiencies.

## Factors associated with protein quality

The human body digests consumed protein into amino acids that are used to build and repair the body protein [9]. Protein sources vary in their amino acid profiles. Although some protein sources contain all indispensable amino acids (IAAs), some contain fewer IAA. Protein quality is described by the crude protein content, the number of IAA, and their digestibility. High-quality protein sources will have high crude protein levels, diverse IAA content, and high digestibility. Therefore, a high-quality protein source will provide people with an adequate ratio of amino acids needed to sustain the body [9]. Social determinants of health and other sociocultural factors (e.g., gender, religious, and cultural norms) may influence a family’s ability to access high-quality protein sources. It is pertinent to understand how protein quality is measured before delving into techniques to improve protein quality.

### Protein requirement, crude protein, and indispensable amino acid content

Overall protein content and amino acid profile in a diet can impact growth and body composition [10]. Proteins are constituted by 20 amino acids, including 9 IAAs, also known as nutritionally essential amino acids, which are not synthesized in the human body at all, or in quantities sufficient to meet the metabolic demand [11]. IAAs must be obtained exogenously through diet because humans lack the genetic code to synthesize some IAAs (histidine, isoleucine, leucine, lysine, methionine, phenylalanine, threonine, tryptophan, and valine) and cannot synthesize enough to meet the metabolic demands in some metabolic states like pregnancy and injury (arginine, cysteine, and tyrosine) [11].

To meet total protein requirements, the recommended intake of protein for the average adult is ∼50 g/d based on the 0.8 g/kg body weight [11], although protein consumption across the globe differs. This estimate is based on an average body weight of 65 kg, according to FAO reference male weight [12]. In 2015, a study reported a global consumption of protein (78.2 g/d) over a third >50 g per day recommendation [11,13]. In contrast, the daily protein consumption varies among LMIC, where the amount of protein consumed per day is lower compared with Western countries, especially for animal protein sources [14,15]. It is also important to note that protein quantity requirements may vary with activity level when food consumption is low [11]. People with lower activity levels may require more crude protein than those with higher activity levels because overall food consumption positively correlates with activity level [11]. Furthermore, a study in LMIC showed that adequate crude protein intake may still represent a protein quality inadequacy [16]. This means that although the population consumes the recommended grams of protein on average, each person may not consume every IAA in the correct ratio depending on the IAA content of the protein source.

For instance, IAAs are abundant in conventional meat and other animal source protein [17]. On the other hand, many plants do not contain sufficient amounts of all 9 IAAs and therefore do not qualify as complete protein sources. In Western countries, ∼60% or more of the total protein consumed by adults is derived from animal-based sources, of which ∼50% comes from meat and dairy [18]. In these settings, shifting toward a more plant-based diet (animal-to-plant protein ratio toward 50:50 or 40:60) is recommended as livestock production and management pose a serious climatic burden [18]. However, when diets include predominantly plant protein sources, which is the case in LMIC, intake of lysine, methionine, leucine, and several other IAAs may become compromised due to the lower IAA content [19]. A deficiency in even 1 IAA is a limiting factor that prevents the body from synthesizing proteins for growth and maintenance [20]. Therefore, populations with plant-based diets may overcome low intake of IAAs by complementing with multiple plant proteins in 1 meal, supplementing, or through considering other traditional or novel methods to improve the quality and digestibility of protein, mentioned in the following sections.

### Digestibility of protein measured using PDCAAS and DIAAS

Protein quality is described by its respective IAA content, the ability of protein to be digested, and the IAA absorption. Digestibility refers to the proportion of macronutrients which are reduced to monomers (amino acids) and are increasingly available for absorption by the gastrointestinal tract [21]. The 2 most common scores used to assess protein digestibility are the Protein Digestibility- Corrected Amino Acid Score (PDCAAS) and Digestible Indispensable Amino Acid Score (DIAAS). The WHO and the United Nations established the PDCAAS metric in 1993 [11] and the DIAAS metric was proposed in 2013 to potentially replace PDCAAS [22]. Both metrics compute scores by comparing the content of each IAA in proteins to age-specific reference IAA profiles to meet IAA requirements and correct the score by the digestibility [23].

PDCAAS uses the fecal digestibility of the protein whereas DIAAS uses the specific ileal digestibility of each IAA [23]. The DIAAS is currently the standard methodology for evaluating protein digestibility because the DIAAS addresses concerns raised about the PDCAAS methodology, including issues regarding accuracy in true fecal digestibility to represent amino acid digestibility [22,23].

According to both the PDCAAS and DIAAS methods, for a given protein source, scores below 1.0 indicate a limited IAAs content within that protein. A total score of ≥1.0 indicates that a protein provides 100% of the required IAAs. For PDCAAS, the score is truncated to 1.0. Protein sources with a score of 1.0 include animal-based proteins (that is, casein, whey, and egg) and some plant-based proteins like isolated soy protein [24]. Other plant protein PDCAAS include 0.9 for pea protein, 0.9 for mustard flour, 0.6 for rolled oats, and 0.65 for cooked beans [24,25]. To improve the protein quality, different plant protein sources can be combined to complement the IAAs profile. Thus, if 1 plant protein source lacks 1 IAA in which another plant protein compensates, then the 2 plant protein sources can be combined in 1 meal or product such that all IAAs are adequately included [26]. For example, cereals and beans are commonly combined to maximize IAAs content [26].

### Antinutritional factors in staple crops

Antinutritional factors refer to biologic constituents naturally found in foods. Antinutrients can have positive effects in food but often negatively impact metabolic performance. Antinutritional factors in staple crops can reduce the efficiency of protein digestibility and nutrients absorption (Table 1) [[27], [28], [29], [30], [31], [32]]. For example, trypsin inhibitors in legumes and grains act as a defense mechanism against insects and other pests though decrease protein digestibility (≤50%) when ingested by humans, although trypsin inhibitors may be inactivated through minimal processing [27,[33], [34], [35]]. Rye and barley contain higher levels of trypsin inhibitors compared with oat and wheat, all of which are common staple crops in LMIC [36]. In addition, lectins are found in legumes and whole grains and may interfere with the absorption of metal micronutrients including calcium, iron, phosphorus, and zinc [37], whereas phytic acid, found in grains, legumes, tubers, and some nuts, which decreases the absorption of iron, zinc, magnesium, and calcium [38]. Phytic acid forms complexes with mineral ions, known as phytates (e.g. inositol-5-phosphate, and inositol-4-phosphate), which makes some positively-charged ions (iron, zinc, etc.) unavailable for digestion [37]. Phytates also decrease protein digestibility by ∼10%–15% [27]. Furthermore, oxalic acid, found in beans, nuts, and beats, binds to calcium and may prevent calcium from being absorbed [37]. Saponins are another antinutrient found in legumes and whole grains that may interfere with normal nutrient absorption [39]. Finally, tannins in legumes decrease iron absorption and protein digestibility with a reduction of ≤23% [27,39]. Several simple processing methods can be applied to mitigate or inactivate these antinutritional factors (Table 1).

**TABLE 1:** Antinutritional factors and simple processing methods that can be applied to mitigate or inactivate these antinutritional factors

Antinutritional factor	Interference with nutrition	Prevalence in staple crops	Mitigation strategies
Trypsin inhibitors	Up to 50% decrease protein digestibility [27,[33], [34], [35]]	Rye and barley [36]	Inactivated by minimal thermal processing (cooking, boiling, roasting) [27,[33], [34], [35]]
Lectins	Interfere with the absorption of metal micronutrients [37]	Legumes and whole grains [37]	Inactivated by high heat [28]. Soaking and boiling can reduce lectin levels [29]
Phytic acid	10%–15% decrease in protein digestibility; decreases the absorption of iron, zinc, magnesium, and calcium [27,38]	Grains, legumes, tubers, and some nuts [38]	Reduced through fermentation, soaking, germination, and enzymatic treatment with phytase enzyme [38]
Oxalic acid	Binds to calcium and may prevent calcium from being absorbed [37]	Beans, nuts, and beets [37]	Boiling and steaming reduced oxalic content [30]
Saponins	May interfere with normal nutrient absorption [39]	Legumes and whole grains [39]	Resistant to heating, but can be washed or grinded off to reduce content [31]
Tannins	23% decreased protein digestibility and reduced iron absorption [27,39]	Legumes [27,39]	Boiling can reduce tannin content [32]

## Diets and protein sources in LMIC

Globally, most plant-based protein in the diet in LMIC comes from staple crops such as maize, rice, wheat, soy, and starchy roots. Plant-based protein intake can also come from legumes and pulses, such as beans or chickpeas, but global consumption of these is less common—pulses contribute 2% to average global caloric intake, compared with 49% by cereals and starches [4]. Diets in which a majority of protein comes from cereals and roots, which have PDCAAS scores ranging from 0.20 to 0.87, have been linked to a high incidence of protein deficiency consequences such as kwashiorkor or stunting in children [16,40,41]. It should be noted that kwashiorkor and stunting may be caused by other factors in addition to protein inadequacy [5].

To avoid protein deficiency and inadequacy, high-quality protein from diverse sources is needed for LMIC populations. high-quality protein sources in LMIC include ASF, such as meat, fish, eggs, and dairy [16]. Consumption of ASF has been shown to benefit the growth and cognitive outcomes of children in LMIC. Studies in Ecuador and Kenya found that eating small amounts of ASF prevented stunting and increased academic and cognitive ability in children, however, a study in Malawi found that supplementation of ASF (especially soft-boned fish) had no impact on height or weight gain [6,7,42]. Livestock keeping is also a major supporter of rural household livelihoods and domestic trade [43]. Livestock rearing can also make use of less-arable land; 57% of grasslands being used to grow animal feed are otherwise unable to grow crops [44]. However, several factors complicate the ability for some to consume livestock as high-quality protein in the diet. Some LMIC may not be suitable for raising livestock; for example, West Africa is unlikely to support cattle due to the risk of tsetse fly infestation [45]. Central and South America, where cattle consumption is high, face the consequences of unsustainable land, water, and energy usage, and high greenhouse gas emissions [46]. From the health perspective, high consumption of red meat like beef, as opposed to other ASFs, such as dairy and eggs, also increases risk of ischemic heart disease [47]. LMIC culture is also relevant; for instance, in some regions of Africa, cattle are not necessarily seen as a food source but rather signs of affluence, such as in their usage as bride prices [48]. In addition, FAO predicts decreased fish consumption in Africa, especially sub-Saharan Africa, due to increased population growth [4]. Thus, it is important to consider alternative approaches to include a diversity of protein sources when possible and increase quality protein consumption in LMIC. In the following section, the global protein intake and plant protein sources are reviewed considering regions, such as sub-Saharan Africa, Southeast Asia, and Central and South America.

### Diets and protein sources in sub-Saharan Africa

**FIGURE 1:**
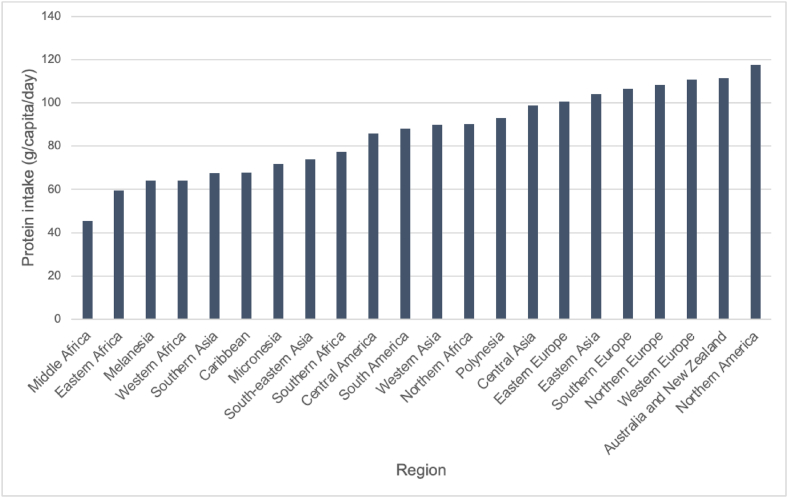
Differences in global daily per capita protein intake, by region. Levels of protein intake, in g/capita/d, compared among different global regions. Data adapted from 2020 FAO food balance sheets [48].

Populations in sub-Saharan Africa mainly consume cereals, such as maize, rice, wheat, sorghum, and millets, and starchy roots like cassava, potatoes, and sweet potatoes. According to FAO food balance sheets, consumption of these cereals and starches is responsible for nearly two-thirds of daily per capita caloric intake [48] (Figure 1, Table 2). Western, Eastern, and Central Africa have the lowest protein intake per capita and highest proportions of plant-derived protein intake (76%, 75%, and 70% respectively) [49]. Eastern and Southern Africa mainly consume maize as a staple crop, whereas Western Africa relies on rice and a variety of other cereals, such as pulses and sorghum [4,49]. Grains are often eaten in bread, grits, soups, milled into flour, or cooked as standalone dishes [50]. Maize is also commonly eaten in an immature state, called “green maize,” which is high-sugar and lower in nutrients compared with mature maize [51].

**TABLE 2:** Sources of staple crop protein intake in Africa, by region^1^

Region	Total protein intake (g/capita/d)	Plant protein intake (g/capita/d)	Staple crop	Staple protein intake (g/capita/d)	%
Middle Africa	45.43	31.98	Cassava and products Maize and products Wheat and products Beans Rice and products	6.59 6.55 3.40 3.24 2.64	20.60 20.50 10.60 10.10 8.26
Western Africa	64.10	49.01	Rice and products Maize and products Pulses, other, and products Sorghum and products Wheat and products	8.05 7.47 6.66 6.09 5.23	16.4 15.2 13.6 12.4 10.7
Eastern Africa	59.67	44.37	Maize and products Wheat and products Beans Pulses, other, and products Rice and products	13.33 5.77 4.95 4.35 3.56	30.0 13.0 11.2 9.8 8.0
Southern Africa	77.38	39.99	Maize and products Wheat and products Rice and products Potatoes and products Groundnuts	19.04 14.15 2.42 1.32 0.54	47.6 35.4 6.1 3.3 1.4
Northern Africa	90.21	58.61	Wheat and products Maize and products Sorghum and products Pulses, other and products Rice and products	34.38 8.32 3.58 3.44 3.04	58.7 14.2 6.1 5.87 5.19

1 Adapted from the FAO 2020 Food Balance Sheet [48].

The main limiting factor contributing to protein deficiency and inadequacy in sub-Saharan Africa is a lack of IAA, particularly those which are lacking in cereals. Maize is very poor in lysine and tryptophan, rice is deficient in lysine, and cassava is deficient in lysine and leucine [[51], [52], [53]]. Starchy foods are low in the sulfur-containing amino acids methionine and cysteine [54]. Because lysine is deficient in all cereals, lysine is the primary limiting IAA in Africa [54,55]. For example, 1 survey estimated that 33% of households in Malawi lacked adequate lysine supply by comparing the household supply of amino acids per adult male equivalent with the estimated average requirements reported in the FAO amino acid profiles [55].

High-quality protein sources in sub-Saharan Africa include milk, meat, and eggs, in decreasing order of per capita consumption [45]. Some significant nonlivestock sources of animal protein in Africa include wild animals (commonly called bushmeat) and fisheries [45,56]. For example, small animals such as the grasscutter, also known as the greater cane rat or *Thyronomys swinderianus*, are considered a delicacy and have spurred their own breeding industry in West African countries, such as Benin, Ghana, and Nigeria [45]. Insects are also traditionally consumed by many rural and urban communities, such as the mopane worm, which are caterpillars of the emperor moth *Gonimbrasia belina* [45]. Fish is particularly important on the West African coast, where it can account for >50% of daily protein intake in the poorest countries including the Gambia, Sierra Leone, and Ghana [57].

### Diets and protein sources in Latin America

Currently, the main staple crop in Latin America is wheat, with various legumes eaten in all parts of the region [58] (Table 3). A study in 2015 by the Estudio Latinoamericano de Nutrición y Salud (ELANS) found that, in 8 Latin American countries, ∼65% of daily protein was animal-based whereas 35% was plant-based [46]. The plant-based protein groups with the highest contribution to protein consumption were grains, legumes, then nuts, and seeds. Most participants in the study that had 9218 participants had an adequate intake of IAAs; the percentage of participants considered deficient in an IAA ranged ≤8% for phenylalanine. The 5 most commonly deficient IAAs included phenylalanine, valine, leucine, lysine, and methionine compared with the FAO standard amino acid requirement metric [46].

**TABLE 3:** Sources of staple crop protein intake in Latin America, by region^1^

Region	Total protein intake (g/capita/d)	Plant protein intake (g/capita/d)	Staple crop	Staple protein intake (g/capita/d)	%
Caribbean	67.93	38.99	Rice and products Wheat and products Pulses and products Maize and products Potatoes and products	9.74 9.45 6.79 3.89 0.39	24.98 24.24 17.41 9.98 1.00
Central America	85.95	45.49	Maize and products Wheat and products Pulses and products Rice and products Potatoes and products	23.65 6.68 5.84 2.24 0.57	51.99 14.68 12.84 4.92 1.25
South America	89.85	38.69	Wheat and products Pulses and products Rice and products Maize and products Potatoes and products	12.10 6.07 5.29 5.28 2.06	31.27 15.69 13.67 13.65 5.32

1 Adapted from the FAO 2020 Food Balance Sheet [48].

### Diets and protein sources in Southeast Asia

Some countries in Southeast Asia similarly derive a high amount of protein from plant protein and experience protein deficiency and inadequacy (Table 4). The main staples in Southeast Asia are cereals, most prominently rice. Since the 1960s, protein availability throughout Southeast Asia has increased significantly due to the expansion of the agricultural sector and an increase in animal protein consumption. Protein consumption in Southeast Asia increased by 85.1% from 1961 to 2018 [59]. As of 2018, most countries, except for Brunei and Malaysia, still consume most of their protein from plants. Daily protein consumption by weight in Southeast Asia exceeds 1 g/kg/d, which meets the requirements, but it does not always meet the requirements for developing children under 5. Although countries such as Malaysia, Singapore, and Thailand meet adequate nutritional requirements, countries with a particularly high rate of childhood stunting include Indonesia, the Philippines, Vietnam, Myanmar, and Cambodia [60].

**TABLE 4:** Sources of staple crop protein intake in Asia, by region^1^

Region	Total protein intake (g/capita/d)	Plant protein intake (g/capita/d)	Staple crop	Staple protein intake (g/capita/d)	%
Southern Asia	67.57	50.10	Wheat and products Rice and products Pulses, other, and products Maize and products Potatoes and products	17.41 12.69 6.89 1.91 1.21	34.75 25.33 13.75 3.81 2.42
South-Eastern Asia	74.08	43.71	Rice and products Wheat and products Maize and products Pulses, other, and products Cassava and products	22.94 5.45 3.59 1.64 0.55	52.48 12.47 8.21 3.75 1.26
Western Asia	89.85	58.09	Wheat and products Pulses, other, and products Rice and products Maize and products Potatoes and products	34.03 5.11 3.99 2.47 1.44	55.32 8.31 6.49 4.02 2.34
Central Asia	98.88	52.90	Wheat and products Potatoes and products Pulses, other, and products Rice and products Maize and products	34.61 3.63 1.76 1.39 1.04	65.43 6.86 3.33 2.63 1.97
Eastern Asia	104.10	61.51	Wheat and products Rice and products Potatoes and products Maize and products Pulses, other and products	17.11 15.12 2.00 1.19 0.97	27.82 24.58 3.25 1.93 1.58

1 Adapted from the FAO 2020 Food Balance Sheet [48].

The main nutrient deficiencies in the Southeast Asian diet are lysine (which is low in rice) and zinc [60]. One plant-based protein which sets Southeast Asia apart from other regions is soy protein, which is highly consumed. Soybeans have one of the highest protein concentrations of any plant product, at 36.5%, and are rich in lysine, making them a good candidate for supplementing cereal-based diets and preventing protein deficiency and inadequacy because soybeans are in addition, a commonly processed plant-based protein [61]. Soybeans are a commonly processed plant-based protein, using techniques described below.

Overall, protein and specific amino acid deficiencies can be prevented by diversifying existing dietary patterns and protein sources consumed in the countries described above and by implementing various techniques that increase protein quality and digestibility.

## Techniques to improve the nutritional quality of local sources of protein

The techniques described below range from techniques that can be implemented at home, such as soaking, cooking, germination, and fermentation, to methods that are implemented on a larger industrial scale due to machinery constraints such as milling, extrusion, drum and spray drying (Table 5).

**TABLE 5:** Summary of the advantages and disadvantages of various methods to improve food quality

Technique	Advantages	Disadvantages
Soaking	Less expensive, less time consuming, low-energy cost	Can introduce contamination [144]
Cooking	Less expensive, low-energy cost, reduces contaminants [143]	Loss of nutrients during heating [65]
Germination	Less expensive, low-energy cost	Germination conditions can introduce bacterial growth [145]
Milling and debranning	Reduces mycotoxins and contaminants [149,150]	Loss of minerals and prebiotic fibers during processing [76,77]
Fermentation	Less expensive, low-energy cost, degrades lactose and cleaning and cooking steps reduce mycotoxins, aflatoxin toxicity, and naturally occurring cyanogens [151]	Products are vulnerable to contamination [153]
Precision fermentation	Reduces allergic reactions [85]	High cost of production due to cultivation and complicated extraction processes [84]
Enzyme addition	Can be done in homes, consumption of protein with foods rich in proteases is less expensive	
Autoclaving	Reduces allergic reactions [139]	High energy cost, can cause Maillard reactions [127]
Extrusion	Reduces mycotoxins and contamination [146,147]	Significant start-up costs, can cause Maillard reactions [126]
Drum and spray drying	Reduces microbial contamination [148]	High energy cost, can cause Maillard reactions [125]
Fortification	Less expensive, can be easily implemented into existing markets [55]	Low-income populations less likely to afford fortified foods [55]
Biofortification	Easy to implement into existing farming practices and local cuisines, can be custom-tailored toward different regions, climates, and consumer preferences [157]	Significant start-up costs, anti-GMO policies and consumer stigma, encourages monopolization and price gouging by seed companies, can have less competitive product appeal compared with existing hybrid varieties [[155], [156], [157]]

### Soaking and cooking

Soaking beans, lentils, and grains often reduces cooking time by making the food softer and enhancing the natural release of enzymes which allows for increased protein absorption due to the breakdown of macronutrients [39]. Soaking is often combined with other techniques and is used as the first step of germination and fermentation [62,63]. Many antinutrients and protease inhibitors are found on the outer surface of beans and legumes and are water-soluble. Therefore, soaking allows them to be drawn out of foods before consumption [39,64].

Different cooking methods also help reduce the antinutrient content of legumes. Pressure cooking and boiling lentils have been shown to reduce the amount of tannins, phytates, and trypsin inhibitors leading to increased in vitro digestibility [65]. Cooking and soaking beans with akanwu, an edible potash salt, is beneficial for tenderizing beans and reducing cook times [66,67]. Soaking and boiling or pressure cooking lentils are common traditional processing techniques used in India and other LMIC, and these techniques can be easily implemented in households in LMIC [68]. However, such cooking methods also lead to the loss of micronutrients, such as vitamins A and C (77% and 61%, respectively, upon 15 min of boiling and 46% and 44%, respectively after 3 min of pressure cooking) [65].

### Germination

Germination occurs when dried dormant cells sprout in appropriate conditions (water, oxygen, temperature, and light in some cases). Upon absorption of water, a radicle emerges from the seed, which will eventually form the root of a plant under appropriate conditions. There are 4 stages of germination: imbibition of water, synthesis of hydrolytic enzymes, metabolism of stored products followed by transport of new products, and the emergence of the radicle [62].

Germination improves the protein digestibility of seeds by activating and synthesizing lipases and carbohydrases, such as ɑ-amylase which disrupt the seed’s microstructure to release proteins in the seeds, allowing for increased absorption [69,70]. Activation of proteases hydrolyze seed proteins can modify protein secondary structure and increase protein digestibility and solubility. Di et al. [71] demonstrated that the surface hydrophobicity of sesame protein and the number of free sulfhydryl groups increased significantly upon germination, indicating unfolding of the protein. They show that the in vitro digestibility and solubility of the protein increased upon germination using enzymatic and solubility assays. Germination can also lead to the hydrolysis of phytin, a salt of phytic acid, and lectins to increase the bioavailability of minerals, and degrade protease inhibitors to enhance protein digestibility further [70,72].

Germinated mung beans are commonly consumed in East and Southeast Asia as sprouts, which can be eaten raw or after cooking [73]. Sprouted grains are often added to bread dough and sprouted seeds and nuts can be added to salads, soups, and stews.

### Milling and Debranning

Debranning is a technique used to remove the outer layers of cereal grains (bran) individually, whereas milling removes all the outer layers at once [74]. Typically, debranning is performed first to remove the outermost layer using friction, followed by milling [75]. These processes allow grains to be ground into flour and reduce the antinutrient content that is present in the bran of grains. However, this process also removes important minerals and prebiotic fibers along with the bran. Although Ertop et al. [76] found that debranning and milling of cereals led to a 65% increase in protein digestibility and a significant decrease in phytic acid, they found it also led to a decreased mineral content. Similarly, Heshe et al. [77] demonstrated that milling of wheat bran led to significantly decreased prebiotic fibers even at a low-extraction rate (68%).

Combining different flours during cooking and baking can help restore mineral content while increasing protein digestibility during debranning and milling in an inexpensive manner in LMIC. Amaranth seeds are commonly added to wheat flour to increase the protein and mineral content of bread and are widely grown and consumed in Africa and Asia [78,79].

### Fermentation

Fermentation is a process by which carbohydrates are converted to alcohol or organic acids using microorganisms (usually fungi or bacteria) under anaerobic conditions [39]. Shekib [63] showed that fermentation of legumes and grains led to drastic increases in the in vitro digestibility of proteins (studied by measuring the amino acids of hydrolysate upon proteolytic enzyme addition) which was attributed to the degradation of stored proteins. Fermentation increases protein absorption by modifying protein secondary structure to reduce the α–helix:β-sheet ratio, blocking compounds that inhibit digestive enzymes and producing microbial proteases that degrade and release proteins from their matrices [80,81]. However, fermentation has demonstrated mixed effects in its ability to decrease phenolic compounds and increase essential amino acids [39,81]. Phenolic compounds can lead to increased crosslinking, making the proteins less accessible to enzymes during digestion in the body [81].

Soy-based traditional fermented foods are common in the Asian and African diet. For example, tempeh is a soybean food fermented with a fungus, *Rhizopus oligosporus*, eaten in Indonesia. It contains 40% protein by dry weight and is rich in vitamin B12 due to its bacterial inoculants. Fourteen percent of Indonesia’s annually harvested soybeans goes toward tempeh production [60]. Tauco, a type of fermented soy sauce similar to miso, is also eaten in Indonesia [60]. Miso is a key source of protein in the Japanese diet, where it accounts for ≤25% of protein intake in some rural inland areas [60]. Fermentation is also a common technique used in the preparation of traditional Indian breakfast foods and in Africa as a food preservation technique [82,83]. Fermentation of maize sourdough or ogi increases essential amino acid content due to lysine and methionine synthesis by microbes [51].

### Precision fermentation

Precision fermentation is an emerging biotechnology technique utilizing microbes such as bacteria and yeast to generate specific functional ingredients that can improve protein digestibility and palatability or can generate edible biomass. Most efforts in the development of precision fermentation techniques have been targeted for the alternative protein (AP) industry, where fermentation is carried out in bioreactors that provide optimal conditions for microbial growth [84]. Precision fermentation can be used to generate proteases that can be added to food products to increase protein digestibility. For example, during fermentation to create plant-based milk beverages, pectinases are released by lactic acid bacteria which improve the protein content, digestibility, and amino acid profile [85]. Precision fermentation can also be used to generate animal proteins or complete proteins containing all IAAs without the animal source using genetically engineered yeast strains [86]. These novel proteins can be added to familiar dishes in LMIC [85].

### Enzyme addition

Proteases are enzymes that hydrolyze proteins. Plant proteases have been historically used commercially in meat and dairy processing [87]. As mentioned above, enzymes can increase protein digestibility by disrupting seed microstructure and allowing for increased protein solubility, or hydrolyzing proteins to make them easier to digest and solubilize [70,71].

Phytase is an enzyme that reduces phytate complex content in food by catalyzing the hydrolysis of phytic acid; when phytase is added during food processing it liberates micronutrients, such as iron, zinc and calcium and improves protein digestibility. In addition, endogenous phytase can be activated by soaking, germinating, fermenting, cooking, autoclaving, and extrusion of foods [88]. Phytase could also be added to food products on an industrial scale so that it could be used during the preparation of foods that are rich in phytic acid to aid digestion [89]. It has been found that healthy males whose diets were supplemented with food-grade protease mixes can lead to increased protein absorption, and decreased C-reactive protein levels [90].

Microbial proteases can also be supplemented to animal feed. The addition of proteases to pig diets has been shown to increase protein absorption and increase endogenous protease activity, especially in younger pigs because their digestive and secretory systems are not fully developed [91]. Similarly, the consumption of foods rich in proteases, such as pineapple, papaya, kiwi, and ginger can aid protein absorption from plant-based protein sources [92]. Ginger is also known to stimulate the release of trypsin, a protease, during digestion [93].

### Autoclaving

Autoclaving is a process of heating materials to an elevated temperature under high pressure. Kalpanadevi et al. [94] demonstrated that autoclaving of legumes led to a ∼70% decrease in antinutrients including tannins, phenolics, and phytic acid. Autoclaving can activate phytases and increase acidity which helps break down phytic acid [95]. Kaewtapee et al. [96] found that autoclaving soybeans led to a doubling of ileal digestibility of all IAAs, although not significantly reducing the amino acid content. Legumes can be autoclaved before packaging in factories to increase digestibility upon consumption and cooking, however this technology process is not currently widely available for LMIC.

### Extrusion

Extrusion is a process by which soft raw food materials are pushed through a shaped opening to generate shaped materials [97]. Important factors to consider during the extrusion of materials to generate easily digestible proteins are the temperature, feed ratio, speed, pressure, and moisture content as they can influence protein structure [97,98]. Low-moisture extruded products typically have low-protein content. During extrusion, the mechanical shear forces disrupt protein bodies, allowing for increased food digestibility, and inactivation of antinutritional factors [98]. Extrusion is typically performed on an industrial scale during manufacturing of food products. Extrusion is also widely used in the AP industry to improve the texture of plant-based meat analogs [99].

Mixed methods can be used to improve protein digestibility and increase protein content. For instance, often, cereals and snack foods can be fortified with legumes to increase the protein content. Patil et al [97] found that while fortifying snacks with legumes, in a process known as food-to-food fortification, only slightly increased its protein content. However, using extrusion along with fortification greatly increased protein digestibility from 37% to 62% weight over volume by degrading protein complexes and denaturing proteins which increases protein digestibility. Textured vegetable protein is an extruded soy-based mixture that is high in protein and is commonly used as a meat substitute [100]. Currently, many ready-to-eat snack foods consumed in the United States and United Kingdom are extruded, and the extruded snacks market has expanded in the Middle East and Africa [101]. Extruded products have long shelf lives and can help combat protein insufficiencies in LMIC [102].

### Drum and spray drying

Drum drying is a dehydration method used to turn liquids, slurries, or purees into flakes and powders by spreading them onto the outer surface of rotating cylinders that are heated by steam [103]. Spray drying uses hot air to remove water from a solution to generate a powder [104]. These processes remove moisture from products which allows them to have an increased shelf life and allows for easy transportation [105].

Various drying methods are used for the dehydration of seaweed and other algae. Drum drying is a low-cost and low–energy-intensive method for drying algae, and leads to dried products with higher digestibility compared with spray drying [106].

In most countries in sub-Saharan Africa and South Asia, children infrequently consume eggs because of egg prices and low availability. Eggs are difficult to import because of their fragile and perishable nature [107]. Spray-dried eggs can be added to plant-based foods to improve their protein and nutritional content. They can be used as a nutrition supplement in LMIC and be provided at lower cost than fresh eggs due to lower storage requirements, ease of transport and longer shelf life [105]. Spray-drying eggs does not lead to significant loss of important vitamins (except for 14% reduction of vitamin A) and only causes minimal loss of IAA content (4%–10%) [105,108].

### Fortification

Fortification is the practice of adding macro- and micronutrients to commonly eaten foods during processing to improve the nutritional quality of a population’s food supply [109]. Adding amino acids to cereal flours is a promising way to prevent protein inadequacy in a high-cereal diet. In China and Syria, researchers reported reduced diarrheal morbidity and improved nutritional markers upon fortifying wheat flour with lysine [110,111]. However, certain populations in LMIC live in rural areas, with greater distance from markets, and will not often purchase commercially available refined foods that would be fortified [55]. Therefore, other mechanisms, such as biofortification, may be a better solution than fortification for populations that are more likely to be small subsistence farmers [55].

### Biofortification

Biofortification involves increasing the nutrient content of a crop through conventional breeding, genetic engineering, and mutagenesis [112]. Other agronomic approaches have been studied, such as addition of nitrogen fertilizer to the soil that crops are grown in which can increase protein concentration in crops because nitrogen is commonly the most limiting factor during crop growth. However, addition of nitrogen fertilizer has been shown to alter the amino acid composition of the crop, specifically lowering lysine and tryptophan, which are IAA, so the level of nitrogen must be finely tuned [113]. Much of biofortification research has aimed to increase provitamin A carotenoids, iron, and zinc content, as these are highly prevalent nutrient deficiencies in LMIC. A secondary goal has been to reduce the inhibitory effect of antinutrient factors, such as phytate that reduce the absorption of these nutrients, either by reducing phytate content or increasing phytase activity [112]. Biofortified crops that specifically increase amino acid content have included maize, cassava, and rice, as well as sorghum and wheat. Below we review 3 examples of biofortification.

#### Biofortification of quality protein maize

Quality protein maize (QPM) is a biofortified maize that is already available in LMIC markets. It contains twice as much lysine and tryptophan as regular maize [114]. QPM was developed through conventional breeding by Surinder Vasal and Evangelina Villegas at the International Maize and Wheat Improvement Center [114]. The mutations initially lowered crop yields, produced a soft, chalky kernel that was unattractive to maize growers, and increased crop susceptibility to drying, ear rot, and infestation. Once a crop with better agronomic characteristics was achieved, international funding for QPM promotion in Ghana began in the 1990s. QPM has since been successfully promoted in Brazil, China, Mexico, and throughout Africa [114]. Various studies in Ghana and Ethiopia reported that children with kwashiorkor responded positively to a QPM diet because consumption of QPM provides 40% more protein than non-biofortified maize [115,116]. Meta-analysis studies have shown that consumption of QPM instead of maize leads to a 12% and 9% increase in rate of growth in weight and height respectively in infants and young children [117].

#### Biofortification of Cassava

Cassava has 1 of the lowest protein contents among the world’s major crops (1.5 mg protein per 100 g fresh cassava) [118,119]. Because of its already low-protein levels, once it is processed into a pure starch, it is completely protein-free. Cassava often needs to be boiled for several hours or fermented because it is a member of the *Euphorbiacean* family, which produces cyanide upon plant injury [52]. Precursors to cyanide, such as linamarin, are also the sources of amino acids for protein synthesis. Therefore, increasing protein synthesis also requires controlled cyanide assimilation. This has been challenging, but researchers are exploring “push-and-pull” methods that increase linamarin conversion (the “push”) and provide a sink to safely store its products (the “pull”) [52]. Another strategy is to express storage proteins. Zhang et al. [120] increased the root protein content by genetically engineering cassava with an artificial storage protein, called ASP1, that contains the maximum possible content of essential amino acids.

#### Biofortified rice

Rice contains low levels of vitamin A, iron, and lysine. Genetically engineered varieties with higher provitamin A (such as Golden Rice) and iron have been tested in African markets. However, lysine-fortified rice has not been introduced to consumers. Researchers have developed some varieties of lysine-fortified rice. Long et al. (2013) [121] made genetically engineered rice lines, named HFL1 and HFL2, to express bacterial enzymes that enhanced lysine synthesis and reduced lysine breakdown. Yang et al. [122] then reported their improvement of growth and food intake when fed to mice. Wong et al. [123] created a rice that overexpressed histones, a lysine-rich protein.

## Limitations and considerations

### Cost and availability of technologies

The approaches described above to increase protein digestibility and solubility and decrease antinutrients range from techniques that can be used at home, to techniques that are performed at a larger scale during manufacturing and in laboratories. Techniques, such as soaking, cooking, and fermentation are often used in homes during food preparation and are low cost for usage in households. Techniques such as fermentation and milling can be done on a larger scale setting and are not too expensive. Many of the other techniques described, however, must be used in a large-scale setting and are often more expensive and require specialized machines such as extruders, lyophilizers, spray dryers, and genetic engineering apparatus. These machines often involve high start-up costs and may be difficult to implement in LMIC [124].

### Food safety and nutritional considerations

#### Maillard reactions and lower protein metabolism

Spray drying, extrusion, and autoclaving can cause Maillard reactions [[125], [126], [127]]. They are the chemical reactions between the amino acid building blocks of proteins and reducing sugars (sugars with a free aldehyde or ketone group, such as glucose, fructose, galactose, maltose, and lactose) in carbohydrates that occur at high temperatures [128]. Maillard reactions can lower protein digestibility, iron bioavailability, and formation of mutagenic products, and can change the color of the food being processed [125,[128], [129], [130], [131]].

Although it has been reported that healthy males whose diets were supplemented with food-grade protease mixes can lead to increased protein absorption, Oben et al. [90] in 2008 found that further research must be done because the researchers observed decreased C-reactive protein levels which could contribute to lower whole-body protein metabolism [90].

#### Allergic or intolerance reactions and insensitivity responses

Allergic reactions to some protein sources may limit their applicability to some populations, though more research is required for LMIC. Allergies exist for certain plant-based proteins, particularly in legumes and nuts. The plant proteins with the highest allergy prevalence include peanuts, soybeans, lentils, chickpeas, and mung beans [132]. Peanut allergies can cause life-threatening symptoms for some with the allergy [133]. Soybean allergies affect 0.7% of people worldwide and ≤10% of people with existing cow milk allergies in Western European countries [134]. Animal products also provide protein sources to LMIC and may cause allergic reactions in some cases. Milk is a common food staple in some LMIC, and cow milk allergy affects 0.25%–4.9% of people globally [135]. Besides allergies, many adults do not easily digest cow milk. Lactose intolerance impacts 68% of the global population, particularly in LMIC [136]. Although seafood can provide additional protein sources in LMIC, people can have fish or shellfish allergies [137]. Animal meat is a common source of protein globally, but its consumption varies in LMIC. Meat allergies are very uncommon, but are possible from alpha-gal syndrome, in which a bite from the lone-star tick and potentially other tick species initiates an allergic reaction to alpha-gal-containing foods [138]. Some techniques described above may reduce allergens in foods. Autoclaving reduces allergic reactions to lentils and chickpeas [139], whereas fermenting milk can degrade much of the lactose out of the final product [140].

#### Contamination and toxins

Contamination and toxins found in plant-based protein sources also pose health concerns in LMIC as food staples. Green leafy vegetables, soft fruits, and sprouted vegetables account for most crop contamination [141]. Human contamination of the environment has led to heavy metals in the soil and uptake into plant crops [142]. Arsenic and cadmium are examples of heavy metals that may affect plant protein quality [142]. Generally, cooking procedures that remove fat from the food will reduce organic contaminants [143]. Soaking can increase contamination by introducing moisture to food, which produces a more favorable environment for microbes [144]. Germinating foods increases the risk of contamination and development of food-borne illnesses because germination typically involves incubation at high humidity at room temperatures that are ideal for bacterial growth [145]. Contamination caused by soaking or germination can be reduced by decreasing the temperature of the water, decreasing the soak time, and increasing salinity [144,145].

Several techniques described in this review can improve food safety by reducing contamination and toxins in food. Extrusion can reduce mycotoxins and reduce contamination in products [146,147]. Spray-drying hinders microbial contamination because the removed water creates a less favorable environment for bacteria to thrive in [148]. Debranning decreases toxins like mycotoxins and decreases contamination levels in wheat and small grains [149,150]. Fermentation includes steps in cleaning and cooking that reduce mycotoxins that exist in some raw materials (cereals and legumes), reduces aflatoxin toxicity in peanuts, and reduces naturally occurring cyanogens in cassava [151]. Biofortified genetically modified organism (GMO) crops may reduce the amount of pesticides inputs on raw materials [152]. Products that are fermented can be vulnerable to contamination, especially meat and dairy-based fermented products [153]. Further research is necessary to optimize fermentation, particularly in the selection of specific starter cultures and pre- and post-processing treatments [153].

### Consumer and farmer preferences and legislation

Despite all the methods to improve protein quality, consumer and farmer acceptance and preference play a major role in whether the protein-rich product gets purchased, grown, or consumed. Consumer preferences and perceptions can affect their intake of nutritious foods. For instance, consumers in Africa tend to prefer more refined flour, whereas whole kernel flour, called dona in Tanzania, is considered “food for the poor” even though it is more nutritious [154]. Increasing consumer awareness about the ingredients and products that have improved nutritional content could help combat these stigmas.

Widespread adoption of biofortified crops in regions, such as Africa has also been slowed due to lack of consumer awareness, as well as GMO policies, and farmers’ preferences for other crops, causing challenges for this mechanism to improve quality protein intake for LMIC consumers. Although not all biofortified crops are GMOs, many crops that could prevent protein inadequacy are genetically modified, and these are hindered from widespread use by anti-GMO policies and awareness [155]. Surveys in South Africa, Nigeria, Ghana, and Tanzania reported a low public awareness of the existence of GMOs and how they work [155]. Still consumer support for GMOs remains low in HIC as well and represents an area where further education campaigns are needed. Particular concerns of consumers which are slowing the commercial introduction of GMOs include opposition to biosafety bills, concerns over plant patents, fear of contamination to indigenous varieties, and contract termination by multinational companies [155].

Furthermore, crop development can involve patents from as many as thirty different companies in which intellectual property rights can lead to increased prices, causing farmers to prefer cheaper options. For example, the biofortified Golden Rice, which has increased provitamin A, was not affordable to farmers until companies agreed to forgo their royalties [156]. Biofortified seeds may also be disfavored by farmers for not matching up to the high yield, appealing taste and color, and hardiness of hybrids which have been bred to maximize these qualities [157]. Proposed policy and education could provide solutions to mitigate these challenges.

### Sustainability

The sustainability of food-processing techniques affects the ability to implement them in LMIC. Techniques with expensive energy and resource expenditures will be challenging to implement. The impact of each technique on the environment is another important consideration. Excessive addition of nitrogen fertilizers to soil during crop growth can be harmful to the environment, as a result of nitrogen pollution. Spray drying is effective for food storage but an energy-intensive technique [158]. Extrusion of raw materials requires energy, but it is an overall efficient process because it combines several industrial steps into one [159,160]. Autoclaving, debranning, and fortification will require energy and produce emissions as they are additional industrial steps. The techniques of soaking, cooking, germination, and fermentation do not pose an environmental or resource concern in terms of emissions. These are methods that can be employed in the home with limited water and energy requirements.

## Discussion

Nutrition challenges in LMIC are widespread, including lack of access to affordable, nutritious food, as well as a lack of education to help citizens make nutritious food choices [2]. Plant-based diets in LMIC that are rich in cereals, roots, and legumes can often lead to protein inadequacy due to low-protein digestibility and insufficient essential amino acid availability [1]. Antinutritional factors in plant-based foods also affect absorption of proteins and micronutrients [161]. Successful interventions to combat protein deficiencies improve awareness, along with accessibility of technology and improve protein quality of existing crops, as opposed to introducing new systems that are not financially sustainable and do not cater to the preferences and needs of individuals in LMIC.

Food-processing techniques range from steps that can be implemented with minimal to no technology at home during cooking, such as soaking, germination and fermentation to large-scale methods such as autoclaving, fortification, and extrusion. Limitations with at-home processing techniques are often related to loss of nutrients and possibility of contamination due to lack of standardized methods, whereas the main limitations associated with larger scale techniques are related to high energy, costs and safety [65,76,77,125,145,153].

In addition, to implement these techniques on a wider scale, efforts by LMIC governments, food companies, and producers to improve food supply chains are crucial. Governments should subsidize farmers growing legumes and biofortified crops by making seeds inexpensive and should make the crops accessible to citizens through public distribution systems partnering with private organizations [162]. Government campaigns to increase awareness for diverse diets and healthy food practices in homes are also necessary. This can be done through awareness activities and programs at the level of schools and community settings to improve uptake of plant protein sources. To make healthy foods more affordable, governments may also provide cash transfers to families in need [162]. Centrally processed products may not be equally accessible to all groups across income levels, so particular attention should be paid to crops and technologies that can expand healthy food consumption without significant cost increases to the consumer. Another hurdle is the negative discourse associated with the health impacts of processed foods. These forms of processed and ultraprocessed foods typically contain additives, such as preservatives, emulsifiers, sweeteners, and artificial colors and flavors. The food-processing techniques described in this review summarize techniques that can have positive impact on nutrition, and do not involve any additives that are linked to negative health effects.

This review focused on the role of plant-based proteins in LMIC, but other novel AP sources, such as cultivated meats from animal cells or insect-based products, could also provide sustainable, nutrient-dense protein options for LMIC. Cultivated meat products may allow manufacturers to modulate the macronutrient and protein quality; however, these products are currently inaccessible to much of the world due to cost, infrastructure, and knowledge required for implementation [163]. Insects are high in protein and relatively inexpensive to cultivate, and some insects, such as silkworms have a complete amino acid profile [164]. Some regions in Asia, America and Africa already utilize insects as a staple protein source and it is a viable option for all LMIC [164,165]. The chitin of insects is commonly removed and they are dried and milled. They can be preserved by drying, fermenting or smoking [164]. Furthermore, an overall balanced diet should address both protein and micronutrient adequacy. Some of the previously described food-processing techniques for enhancing protein also increase mineral and vitamin bioavailability, such as fermentation and germination.

In conclusion, on the basis of the techniques reviewed, the recommendation to combat protein deficiencies, using a plant-based diet, is to use as many techniques described above as possible. These techniques can improve protein quality and thus could be used simultaneously to enhance protein quality from local protein sources and primarily plant-based diets. More research will be needed to identify which, if any, techniques are best for supporting improved protein quality in the different regions of LMIC. Future studies should directly compare the effect of multiple techniques on protein digestibility and absorption and consider consumer acceptability and cost of products and evaluate the direct impact of protein quality on nutrition. These approaches will allow communities to utilize existing food sources with few additional processing steps to improve quality of locally available plant protein sources.

## Author contributions

The authors’ responsibilities were as follows – NV, MS-C, SL: conducted research; SL: analyzed FAO data to develop the figure; NV, MS-C, SL: wrote the first draft of the manuscript; SB, WS, DT, RK: provided suggestions that resulted in subsequent drafts; DT: led the submission process; and all authors: read and approved the final manuscript.

## Conflict of interest

The authors report no conflicts of interest.

## Funding

NV, MSC, and SL are voluntarily involved with the Good Food Institute through the Alternative Protein Project at Johns Hopkins with no funding received.

## Data availability

Data described in the manuscript, code book, and analytic code will be made available upon request.
